# The Relative Importance of Various Subjects in Practical Sanitation

**Published:** 1882-03

**Authors:** 


### POPULAR SCIENCE DEPARTMENT.

### THE RELATIVE IMPORTANCE OF VARIOUS SUBJECTS IN PRACTICAL SANITATION.

DR. C. B. WHITE'S PRESIDENTIAL ADDRESS BEFORE THE AMERICAN PUBLIC HEALTH ASSOCIATION, AT SAVANNAH, GA., NOV. 29, 1881.

(*Concluded from February Number.*)

The experienced and fully informed mind reflects that observations may not have been sufficiently extensive, or not sufficiently extended as to time; that as vital questions involve many factors, some may have been overlooked, or not duly considered; that an important element may be recognizable by consequences which appear at a remote date; that the results of evil practices may be found nearer the end of life, as an early, decrepit old age, instead of an old age, vigorous and late; or of children born of those living in such unfortunate circumstances, thence inherit a feebler organization and low viability.

The sanitarian of broad views looks at the healthy child, with dirt-begrimed skin; but underneath the filth the mind's eye sees a texture secretive, exhalent, extrusive, the exact opposite of an absorbent surface, throwing off poisons from the interior, and practically impassable to poisons from without.

The Sanitarian perceives the air none too savory; his microscopic examination of similar atmospheres has revealed spores, bacteria, particles of animal matter, alive and dead, but he bethinks himself of the arrangements for entangling and returning much of this matter before it reaches the ultimate air cells, and especially of that wonderful lining tissue of the lung, which with chemical precision passes in oxygen and lets out carbonic acid and vapor of water, yet with such certainty retaining the blood upon its other side that one may see a thousand cases of that dread destroyer of texture and blood, yellow fever, and see but a single caseof hemorrhage from the lungs, and even that in one already a consumptive.

Such reflections naturally suggest the widely diverse nature and methods of the hygiene of the individual and race hygiene.

In the shipwreck we see a single person outlasting all others of the company, defying flaming sun and winter's rigor, the pang of hunger and the anguish of thirst; or in the disastrous retreat, those who outlive frost, famine or fever. If a deadly epidemic rage, we see those who altogether resist it, or if attacked recover, despite lack of professional care, violation of the most ordinary rules of prudence, and with every circumstance inimical to recovery. Or we see the subject of some wasting, generally fatal, disease, or the victim of street conflict, or steamboat disaster, or railway accident, groaning "with the groans of a deadly wounded man," yet seemingly finding it impossible to die, and to the amazement of the profession and on-lookers, outlasting all malign prophecies, recovering health, and again doing his part of the world's work.

This limitless endurance, this clenched tenacity of life and enormous viability are at once the evidence and the result of race hygiene.

Nature, by the process of selection, mercilessly weeds out the feeble.

Careful as she would seem in securing abounding numbers of the race, as a whole, she takes no thought of the individual. Nature, too, would seem to exercise small judgment in her severity. The tables of the world's death rates show that in cities more than one-half, and in the country, that nearly one-half born into the world die on or before completing the fifth year of their existence. Physicians remember infants of exceeding feebleness or suffering from long and wasting sickness retaining life even, contrary to all expectation, yet now developed into robust young men, or healthy, strong and comely women.

The hygiene of the individual only, would here, if applied, immensely and immediately decrease this death rate, so shocking to the human mind.

It is impossible to believe that one-half the human race is born non-viable. If it were true, most appropriately this multitude of slain innocents might join in the tombstone soliloquy of the infant buried in the London cemetery, dead at one day old: "If so soon I am done for, I wonder what I was begun for."

These much-enduring disease, death resisting specimens of the human family, before spoken of, show what a succession of fortunately environed generations can develop. The surroundings of these persons may have been altogether highly favorable, but it is the continuance of them through a series of generations which has furnished the result. As education may do much for a man, but what he was in the beginning before he received education, what the product of the generations was, is the main and true reason of what the man becomes. So hygiene of the individual, like his education, has a certain and large value to him, but the "Sanitary Philosopher," while appreciating this and earnestly working for the best hygiene of the present, loves even more to look upon the great result to the human race of the hygiene of the individual maintained during a series of generations. His prophetic eye is filled with pleasant visions of the race in possession of higher health, longer life and greater intelligence, a better aptitude for scientific and literary pursuits, and every avocation of life.

The happy circle of his view shows man with bad passions in control, his pleasures purer, more numerous, more exquisite, and less exhausting.

How fortunate for both the present and future age, did parents appreciate the worth of correct sanitary practices, and wisely think and do for both themselves, their children, and their children's children. Grateful and graphic is the simple Old World picture of a city prosperous and healthy: "There shall yet old men and old women dwell in Jerusalem, and every man with his staff in his hand for very age. And the streets of the city shall be full of boys and girls playing in the streets thereof."

Were the laws of hygiene exactly known, certain isolated individuals, by careful and continuous obedience, might expect to obtain the largest benefits in the way of life, and health and enjoyment, that their own careful effort, and the constitutions and practices of their ancestors permitted, and also to transmit to posterity a still further improved heredity; but, alas! the epidemic diseases forciby intrude themselves, bearing and peremptorily enforcing the lesson that no man liveth unto himself, that personal hygiene is not simple but complex; the interests of the unit and of the sum total of the community, are found to be one; the would-be selfish human monad can neither live or perish to himself, and State medicine is at once a natural outgrowth of human relations and a necessity.

There is nowhere just or general appreciation of the importance which attaches to the position of the représentative of State medicine, Health Officer or Board of Health. To properly exercise their functions, as, for a single example, to deal effectually with epidemic diseases, includes the knowledge of the nature, cause, habitat and habits of such sicknesses, if known, and methods of repression available. They must precisely be informed of what is and what is not known, must have broad views, executive ability and fearlessness of the highest personal and moral order.

The exceptional quality of such labor deserves equally exceptional pay.

To the contrary, I know of but one locality in the United States where those responsible for the public health are paid in such measure as railroad companies, banks, or great mercantile corporations, recompense work of similar high value.

The excuse for a contrary procedure sometimes is, that the work does not require all "the doctor's" time, that there is not much to do, and frequently "the doctor," miscalled sanitarian, thinks so too. To keep well informed in advancing hygiene, without doing any original scientific work, will occupy the time of any man who is practically engaged even a part of the time.

That man is often the best employed, and most overworked sanitarian, who is doing nothing at the moment for the citizens under his care, but who is eagerly widening the too narrow limits of his knowledge, accumulations of which are even better than the domestic "saving up" of articles of no apparent value, for a piece of information seldom waits seven years to come of use.

The man who thinks there is little to do or learn is of that opinion, because he does not know the extent of his ignorance.

The truth is sanitarians go a warfare at their own charges.

Physicians whose livelihood is directly and solely derived from the treatment of the sick have in all time been incessant in discourse and labor for the prevention of sicknesses.

The sanitary work of the world has been done, and to day is being done, for love of knowledge and love of man.

Sanitary science has reached the point that further great advance, its most gainful researches, can be secured only by the expert, who gives all his time to study and practice, and brings to his work intelligence, knowledge and training. I cannot refrain from a moment's mention of a single important service to be expected from this class of practical thinkers. In this country the mass of those interested in, and even of the actual workers in science, are, so to speak, amateurs. We may call them the irregular troops, the volunteers, the free lances of science, possessing an abundance of zeal, with an equally abounding insufficiency of knowledge. To this great number our expert force will be of incalculable value, in the words of Mr. Vernon Harcourt, " the law-giver and proper founder of the British Association for the Advancement of Science," "giving a stronger impulse and a more systematic direction to scientific inquiry; in pointing out the lines of direction in which the researches of science should move; in indicating particulars which most immediately demand investigation; in stating problems to be solved and data to be fixed." *

This class of service, difficult to obtain, now and in the future grown to be essential, must be furnished by the State.

But were this complete arrangement for the hygienic care of the man as an individual and a citizen secured, each State, sovereign though she be, is yet one of a fairly large family. Diseases prevailing in one may threaten another member of the family, and the precautions needful overpass boundary lines and require a higher power unsectional and impartial, to arrange, adjudicate and regulate. This higher health authority, of necessity national, can well receive, arrange, record and publish the vital statistics of the whole country, make researches not within the scope of State power and interest, receive information of the presence, increase or decline of disease throughout the country, or even ultimately to forecast the state of the public health and issue its bulletins of instruction, of warning or of comfort,

The rapid transit of civilization having brought the nations into vicinage, intimacy of intercourse requires international sanitation and national sanitary commissioners.

To maintain what small advantages sanitary science has secured, to obtain the benefits which will accrue from the practical working of the sanitary scheme so curtly sketched, is the important matter press-

---

* It will only occupy a few moments of your time to illustrate by example the material sometimes furnished to men of science from which to draw conclusions. A surgeon of a regiment lately assigned to an army corps, at the close of the month sent in the customary monthly report. As all feeble men had been removed from the regiments preparatory to an active campaign, and the corps lay in a healthy location at a healthy season of the year, the number of the sick and the diseases from which they suffered, attracted the attention of the Medical Director. The next report presented the same peculiar features. The surgeon was sent for and explained: "*I keep no daily record, so that at the end of the month I sit down and think how many have been sick, and then, running my eye along the list of diseases, I pick them out in equal numbers, so as to make an interesting and variegated report.*

ing on us and all sanitarians. One important help to the result will be the establishment of a professional chair and lectures on hygiene in every medical school, attendance upon lectures, and final examination upon that study, being made necessary to graduation. The desired result must, however, be mainly attained in one way—educate the people.

We want immediate benefits, therefore we must do that thing so difficult to effect—educate adults. Legislators and governors must learn.

The press must continuously, pointedly and indefatigably set the truth before its readers. There must be sanitary circulars, and sanitary conventions and sanitary fairs.

The clergy must preach from the texts, "Know ye not that your bodies are the members of Christ?" and to a following scripture, "What! know ye not that your body is the temple of the Holy Ghost?" They must instruct their congregations in the way of keeping that temple meet for the presence and in-dwelling of a Gracious Spirit; they must call aloud for such a life as shall secure that the temples of the future be better and more beautifully builded, and denounce with righteous indignation those who injure, or who shorten the life of that most wonderful existence, the human being, the result of God's ages, the flower of evolution.

Above all, take care for the children!

First, secure the writing, publication and adoption of text-books largely illustrated, suited to different ages. Next, after a specified time, allow no one to teach school, unless so familiar with the fundamental principles of physiology and hygiene, that children learn not only while the book is in the hand, but constantly receive information from an intelligent instructor, with whom maxims of health and pertinent illustrations are as ready for speech, as those of morality and philosophy. In such a system of instruction, errors in physical conduct or idea will be pointed out for amendment, as mistakes in grammar, pronunciation, and behavior are corrected.

Introduce hygiene early in course, that what may be then learned serve as a foundation for continued improvement, as a crystal begun gains constant accretions from the mother liquor.

The scientifically-taught mind, like a coral growth with every polype mouth open, eager to appropriate from the surrounding sea, will be always learning, be it from teacher, book, associate or wide openeyed observations. Before finishing the usual round of studies, a higher grade of book and study should be traversed to take advantage of mental growth and maturity.

Whatever else be left undone or untaught, sharply, deeply and abidingly impress upon the mind of the young, that at every period of existence, how to be healthy is the most important study of every human creature.

The result will be a generation wise in its own interests; its women will do well that largest part of the development of the race, and of the hygiene of the home which is their lot and privilege in life; it will furnish its own experts, whose scientific purposes were kindled and fanned by education; its legislators will be well informed, and therefore favorable to public health interests, and will no longer wish or dare to give to politicians, or necessitous and undeserving friends and relatives all the money needed to preserve the health or save the lives of the people.

Could I to-night lift the roofs of houses and hospitals, and show an uninformed and indifferent public, those now lying sick and dying of preventable disease, or pass before it the graves of the tens of thousands of those killed by avoidable diseases within the year just passed, or accumulate the figures and demonstrate the loss of time and loss of wages and expenses of sickness and death, summing, too, the physical sufferings and anxieties of mind unnecessarily endured, or assemble before this ignorant public the exceeding great multitude of little children—the innocents cruelly slain without cause—and their mourning relatives and friends, the community would rise as one, demand and secure a far-reaching and permanent change. Surely, "the people perish for lack of knowledge."

There exist those who maintain that the world is already too crowded, that if sanitary science keeps alive those who now unnecessarily die, the present struggle for existence, ending in the survival of the fittest, will continually become more fierce, and the bitter results of the battle of life, the unavoidable sufferings of those who go down, and are trodden out of existence, will much more than counterbalance all the blessings which perfected science, carried to completed ends, can create.

Answer enough for this age is to point as illustration to Louisiana's millions of acres, of fathoms deep, black soil—only awaiting sanitary engineering to support a dense, healthful and prosperous population.

The extraordinary progress lately made by men of science gives sure foundation to the belief that in a future not far remote, the struggle for existence will not be so much man's hand to hand contest with his fellow for life and comfort, but will be rather man's contest with the forces of nature, man the victor, with increasing rapidity turning to his own service energies now wasting themselves about him, more amply supplying necessities, steadily augmenting comforts, and relatively diminishing labor and exhaustion. No longer man against man, but all men brethren, made rich from nature's treasuries.

